# Data analysis between controllable variables and the performance of CuS crackle based electrode

**DOI:** 10.1016/j.dib.2018.01.062

**Published:** 2018-01-31

**Authors:** Zijie Xu, Teng Li, Qian Liu, Fayin Zhang, Xiaodan Hong, Shuyao Xie, Changxu Lin, Xiangyang Liu, Wenxi Guo

**Affiliations:** a Research Institute for Soft Matter and Biomimetics, Fujian Provincial Key Laboratory for Soft Functional Materials Research, Department of Physics, Xiamen University, Xiamen, 361005, China; b Department of Physics, Faculty of Science, National University of Singapore, Singapore 117542, Singapore

## Abstract

In this article, we provide the data analysis between controllable variables and the performance of CuS crackle based electrode, there are four important factors which could influence the formation of cracks, the colloid concentration, drying temperature, colloid dosage and ambient humidity. We carried out and summed nineteen controlled data experiments below and other variates which could affect the performance were discussed in this article.

**Specifications Table**

**Table:** 

Subject area	*Chemistry, solar energy*
More specific subject area	*Transparent conductive film, dye-sensitized solar cell (DSSC)*
Type of data	*Table, text file, figure*
How data was acquired	*Microscope, survey, SEM, four-probe arrangement, UV–vis spectroscopy instrument (Lambda 750)*
Data format	*Raw, analyzed*
Experimental factors	*Ultrasonic cleaning, magnetron sputtering, hydrothermal method*
Experimental features	*There are four important steps in fabricating the CuS counter electrode(CE) the first step is to coat the P25 TiO*_*2*_*, it is worth to note that the colloid should be put in vacuum, any airflow would change the uniformity of the crack. Second step is the magnetron sputtering process, notably, the vacuum degree should be as high as possible, Cu is prone to be oxidized when the air exists. The third step is template removal, by mechanical lapping with weak ultrasonic vibration, TiO*_*2*_*template could be easily removed. Last step is sulfuration, it is worth mentioning that S power ethanol solution should be filtered in case the undissolved S power pollutes the sample.*
Data source location	*Xiamen university, Xiamen, China*
Data accessibility	*The relevant data we have provided in the main article and its corresponding supplementary information, besides we have provided other data in this data in brief. If the readers need more data please mail: wxguo@xmu.edu.cn*

**Value of the data**

● The relationship between the colloid concentration and the performance of CuS crackle based electrode (include crack width, transmittance and conductive) was exhibited by controlled experiments.
● The relationship between the colloid dosage and the performance of CuS crackle based electrode (include crack width, transmittance and conductive) was exhibited by controlled experiments.
● The relationship between the drying temperature and the performance of CuS crackle based electrode (include crack width, transmittance and conductive) was also exhibited by controlled experiments.
● Other variates which could affect the performance of CuS crackle based electrode were also displayed in this data in brief.

## 1 Data

**Fig. 1:**
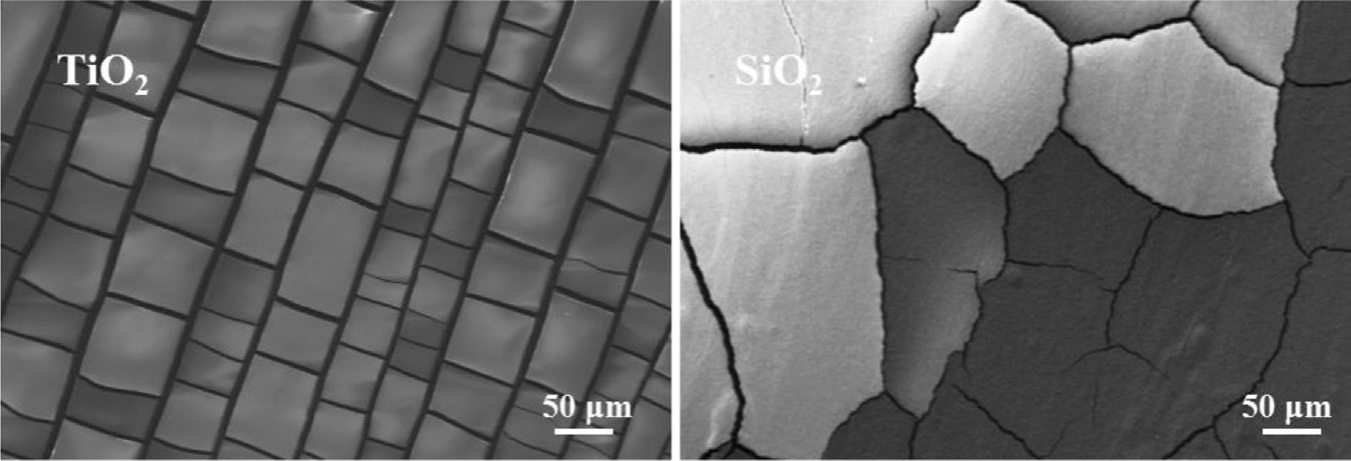
The SEM images of the comparison between SiO_2_ based crack and TiO_2_ based crack.

**Fig. 2:**
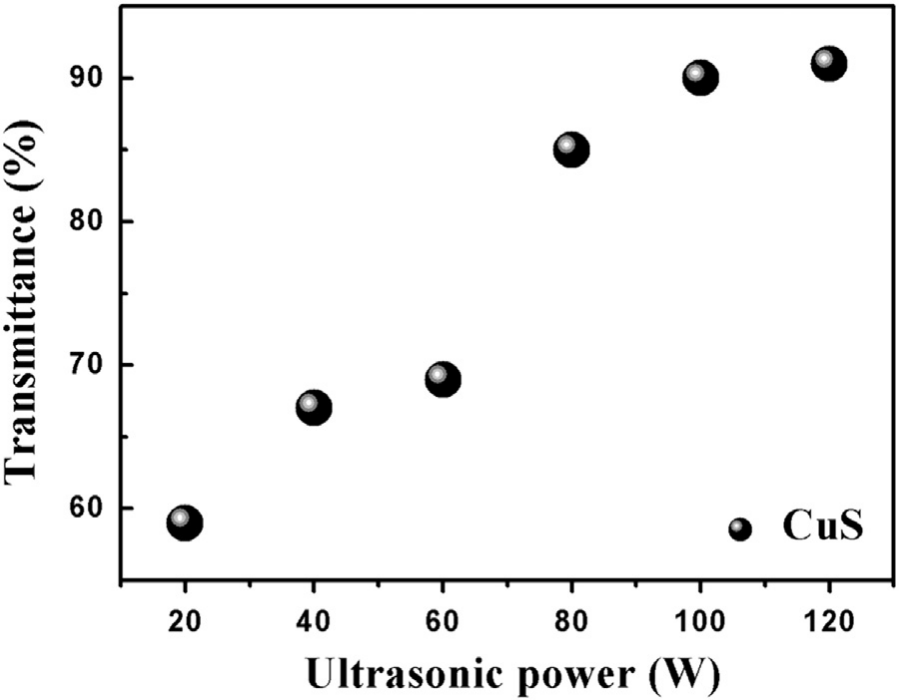
The relationship between ultrasonic power (30 s template removal) and the transmittance of CuS film.

**Fig. 3:**
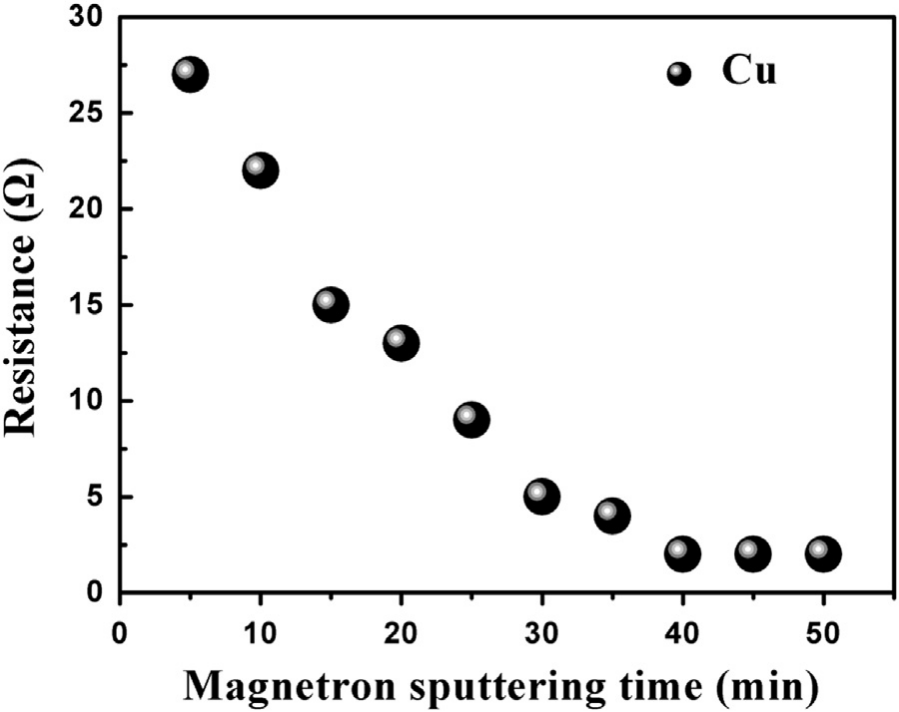
The relationship between magnetron sputtering time (100 W) and the conductivity of Cu film.

**Fig. 4:**
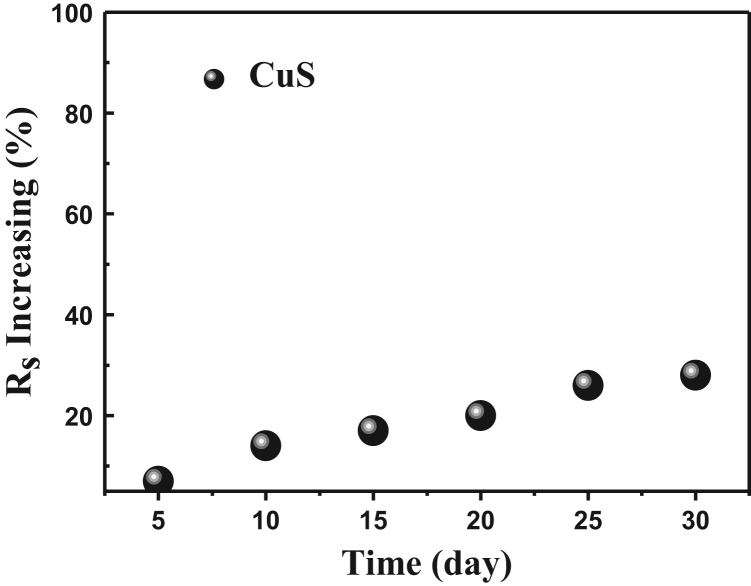
The relationship between the sample placing time and the resistance increasing.

See Table 1 and Fig. 1, Fig. 2, Fig. 3, Fig. 4.

**Table 1:** 19 typical controlled experiments for fabrication of crackle based CuS films.

**Groups**	**Trunk crackle average width (μm)**	**Dosage (μL cm**^**−2**^**)**	**Concentration (g 10 mL**^**−1**^**)**	**Drying temperature (**°C**)**	***R***_**s**_**(Ω sq**^**−1**^**)**	**Transmittance at 550 nm (average) (%)**
1	6	30	1.6	40 (vacuum drying)	58	91
2	16	30	1.8	40 (vacuum drying)	49	85
3	32	30	2.0	40 (vacuum drying)	33	76
4	5	20	1.6	40 (vacuum drying)	66	90
5	11	20	1.8	40 (vacuum drying)	55	87
6	25	20	2.0	40 (vacuum drying)	49	79
7	2	30	1.6	20 (vacuum drying)	51	90
8	10	30	1.6	60 (vacuum drying)	54	86
9	19	30	1.6	80 (vacuum drying)	49	80
10	52	30	2.0	100 (vacuum drying)	36	71
11	65	60	2.0	40 (vacuum drying)	33	71
12	96	60	2.0	80 (vacuum drying)	28	62
13	9	30	1.6	40 (dry in air)	53	90
14	14	30	1.6	60 (dry in air)	46	82
15	22	30	1.6	80 (dry in air)	48	80
16	8	20	1.6	40 (dry in air)	58	86
17	75	60	1.6	40 (dry in air)	37	68
18	19	30	1.8	40 (dry in air)	55	83
19	33	30	2.0	40 (dry in air)	49	78

## 2 Experimental design, materials and methods

The data analysis for controllable variables versus the performance of CuS transparent conducting electrode(TCE) is provided here: first, four important factors could influence the morphology of cracks, they are the colloid concentration, drying temperature, colloid dosage and ambient humidity, respectively [1]. To further investigate the relationship between the four factors and the performance of CuS TCEs, 19 typical controlled experiments listed in Table 1 were carried out, besides, the SEM images of the SiO_2_ based crack and TiO_2_ based crack were displayed in Fig. 1, respectively. TiO_2_ based crack shows greater order property than SiO_2_ based crack in this work. Fig. 2 shows the relationship between ultrasonic power (30 s template remove) and the transmittance of CuS TCEs, along with the increasing power, the transmittance is increasing until it reaches its maximum value (~ 90%). Fig. 3 exhibits the relationship between magnetron sputtering time (100 W) and the conductivity of Cu film. When the sputtering time is 40 min, the sheet resistances (*R*_s_) of Cu film reaches its minimum value. The relationship between the stability testing time in ambient condition and the resistance increasing is shown in Fig. 4, after testing for 30 days, only ~ 30% increasing was observed. These data comparisons were designed to analyse the relationship between controllable variables and the performance of CuS crackle based electrode.
