# Supplementary material for: Data analysis between controllable variables and the performance of CuS crackle based electrode
Source: Data Brief. 2018 Jan 31;17:1331–5. doi: 10.1016/j.dib.2018.01.062 (PMC5988386; doi:10.1016/j.dib.2018.01.062)
Supplement: Supplementary file 1 — Supplementary material [file mmc1.docx]

Conflict of Interest

The authors have declared that they have no conflict of interests.
